# Case Report: Ursodeoxycholic acid treatment in Niemann-Pick disease type C; clinical experience in four cases

**DOI:** 10.12688/wellcomeopenres.11854.1

**Published:** 2017-08-31

**Authors:** William R.H. Evans, Elena-Raluca Nicoli, Raymond Y. Wang, Nina Movsesyan, Frances M. Platt

**Affiliations:** 1 Niemann Pick UK, Tyne and Wear, NE37 2SQ, UK; 2 Department of Pharmacology, University of Oxford, Oxford, OX1 3QT, UK; 3 Division of Metabolic Disorders, CHOC Children’s, Orange, CA, 92868, USA; 4 Department of Pediatrics, University of California-Irvine, Irvine, CA, 92617, USA

**Keywords:** Niemann Pick disease type C, NPC, lysosomal storage disease, treatment, cholestasis, p450

## Abstract

In this case series, we demonstrate that Ursodeoxycholic acid (UDCA) improves liver dysfunction in Niemann-Pick type C (NPC) and may restore a suppressed cytochrome p450 system. NPC disease is a progressive neurodegenerative lysosomal storage disease caused by mutations in either the *NPC1* or *NPC2* genes. Liver disease is a common feature presenting either acutely as cholestatic jaundice in the neonatal period, or in later life as elevated liver enzymes indicative of liver dysfunction. Recently, an imbalance in bile acid synthesis in a mouse model of NPC disease was linked to suppression of the P450 detoxification system and was corrected by UDCA treatment. UDCA (3α, 7β-dihydroxy-5β-cholanic acid), a hydrophilic bile acid, is used to treat various cholestatic disorders. In this report we summarise the findings from four independent cases of NPC, three with abnormal liver enzyme levels at baseline, that were subsequently treated with UDCA. The patients differed in age and clinical features, they all tolerated the drug well, and in those with abnormal liver function, there were significant improvements in their liver enzyme parameters.

## Introduction

Niemann-Pick type C (NPC) is a rare neurodegenerative lysosomal disorder, with a clinical incidence of 1:100,000 ^1– 4^. It is caused by mutations in one of two genes: *NPC1* (chromosome locus 18q11.2), or *NPC2* (chromosome locus 14q24.3), with *NPC1* accounting for 95% of diseased patients ^1, 2^. The exact pathogenesis of NPC remains unclear, but loss of function of either the NPC1 or NPC2 protein leads to the accumulation of cholesterol and glycosphingolipids in the late endosomal/lysosomal compartment. This leads to disrupted lysosomal calcium homeostasis and a host of secondary cellular trafficking defects ^5^.

The disease is highly heterogeneous in both neurological and visceral features. Neonatal cholestasis is a common presenting feature; indeed, NPC is considered the second most common genetic cause of liver disease in infancy after alpha-1-antitrypsin deficiency ^6^. This phase of the disease is usually self-limiting, with resolution of jaundice at 2–4 months of age ^7^, often before an accurate diagnosis is made. A smaller number of patients have more severe early liver disease, with fetal hydrops, ascites and liver failure reported ^1, 7^. Interestingly, the severity of liver disease gives little indication of the age of onset or rate of progression of later neurological disease ^1, 2^.

NPC neurological disease is highly variable, spanning a spectrum of severity and age of onset, but is nearly always progressive. Early-onset patients present as toddlers with delayed motor milestones and hypotonia. Other patients present during their early school years with clumsiness, ataxia, speech and learning difficulties, while juvenile onset patients present with a similar combination of features but in later childhood. A substantial sub-population has adolescent or adult-onset neurological disease with dementia, ataxia and psychiatric features dominating the clinical presentation. Cataplexy and seizures can also be present, especially in the childhood onset cases. The most frequently encountered and clinically specific neurological feature across all ages is a vertical supranuclear gaze palsy (VSGP), which often heralds the onset of neurological disease ^1, 7^. Other bulbar involvement includes dysphagia and aspiration.

Palpable hepatosplenomegaly is identifiable on clinical examination in 85% of NPC patients, but is identified less frequently in those with adult/adolescent onset disease ^2^; however, on abdominal ultrasound it can be detected in 90% of patients regardless of age ^1, 7^. Excluding a limited number of patients with severe NPC-related paediatric liver disease and its consequent sequelae, the persistent hepatosplenomegaly is considered asymptomatic. However, many have on-going mild elevation of transaminases and thrombocytopenia ^1^, suggesting liver function is not normal.

Current NPC treatment is supportive, managing complications of the disease including seizures, cataplexy, ataxia, dysphagia, and orthopaedic manifestations. Miglustat ( *N-*butyldeoxynojirimicin, or Zavesca: Actelion Pharmaceuticals) is the only pharmacotherapy licensed for NPC (though not in the USA). Miglustat is an iminosugar substrate reduction therapy drug ^1^ that reduces the accumulation of toxic substrates, including glycosphingolipids, by competitively inhibiting glucosylceramide synthase, the first and rate-limiting step in glycosphingolipid biosynthesis ^8, 9^. The development of therapies for NPC is extremely active, with several clinical trials in progress, and gene-correction strategies in preclinical development.

### The role of ursodeoxycholic acid (UDCA) in NPC

The potential role of UDCA in the treatment of NPC disease arose from the discovery of suppressed hepatic cytochrome P450 in the *Npc1* null mouse model ( *Npc1 ^-/-^)* ^10^. One of the many consequences in NPC is a deficiency in the efflux of unesterified cholesterol from late endosomes/lysosomes to the endoplasmic reticulum (ER) and Golgi ^11^. The cytochrome P450 enzyme system participates not only in the oxidation and detoxification of xenobiotic compounds, but also the synthesis of bile acids from cholesterol. The resultant imbalance in cholesterol-derived metabolites, especially bile acids (which are synthesized in the ER by various cytochrome P450 enzymes), supresses gene expression and subsequent activity of those P450 system enzymes ^12, 13^. The hepatic cytochrome P450 enzyme deficiency in the *Npc1 ^-/-^* mice was rescued by treating them with UDCA, which also surprisingly resulted in some improvement of neurologic disease ^10^. UDCA, the principle bile acid of the black bear ( *Ursus americanus*), is normally only found in low concentrations (~3%) in human bile ^14^. It is used as a treatment for gallstones and cholestatic liver disease, including primary biliary cirrhosis, cystic fibrosis-associated hepatobiliary disorders, primary sclerosing cholangitis, cholestasis of pregnancy, hepatic graft versus host disease and neonatal cholestasis ^15– 20^. When post-mortem liver samples were studied from NPC1 patients, suppression of the P450 enzyme system was detected similar with those seen in *Npc1 ^-/-^* deficient mice ^10^. These key observations in mice and humans suggested that NPC1, and likely NPC2, patients could also have this key detoxification system restored with UDCA therapy. In this paper, we report our clinical experience of UDCA therapy in the hepatic and neurologic manifestations of four NPC patients ^21^.

## Case 1

A female child of Chinese descent was conceived by a 37 year old G _1_P _1→2_ woman by *in-vitro* fertilization and born at 33 ^+1^ weeks’ gestational age with a birth weight of 1315g (2 ^nd^ percentile, UK-WHO growth charts ^22^). At three weeks of age, acholic stools were noted and she was found to have cholestatic jaundice (total bilirubin 6.9 mg/dL, reference range [RR] <1 mg/dL; direct bilirubin 4.4 mg/dL, RR <0.4 mg/dL), elevated transaminases (AST 159 U/L, RR <63 U/L; ALT 54 U/L, RR <32 U/L) and alkaline phosphatase (422 U/L, RR <321 U/L) with normal γ-glutamyl transpeptidase (33 IU/L, RR <50 IU/L). Coagulation times and blood counts were normal: prothrombin time international ratio was 1.0 (RR <1.2), partial thromboplastin time 37.4 s (RR <40), and platelet count 216 k/µL (RR 150 – 200 k/µL).

Imaging studies included an abdominal ultrasound that demonstrated hepatosplenomegaly and normal hepatobiliary anatomy, as well as a cholescintigraphy scan that failed to identify tracer excretion into the gallbladder. She subsequently underwent an intraoperative cholangiogram that was normal; liver biopsy was also obtained showing severe intercanalicular cholestasis, hepatocyte ballooning with giant cell formation, portal inflammation, and extramedullary hematopoiesis. UDCA 30 mg/kg/day was started for treatment of cholestatic jaundice at age four weeks.

Biochemical investigations ruled out galactosemia, hepatorenal tyrosinemia, fatty acid oxidation disorders, organic acidemias, amino acidopathies, congenital disorders of glycosylation, peroxisomal disorders, disorders of polyol metabolism, mucopolysaccharidosis type VII, oligosaccharidoses, Farber disease, and lysosomal acid lipase deficiency. Of note, her acid lipase activity was above normal range, at 681 nM/punch*hr (RR 79.9 – 378.6 nM/punch*hr). Post-biopsy, she could not tolerate attempts at extubation, and a tracheostomy was placed at five weeks of life. Mechanical ventilation was difficult, initially requiring high positive end-expiratory pressures (PEEP) of +7 cm H _2_O due to atelectasis from hepatosplenomegaly and abdominal distension, and often exacerbated by recurrent bacterial tracheitis. Her direct bilirubin ranged between 4 – 7 mg/dL and total bilirubin between 7 – 10 mg/dL, peaking during episodes of tracheitis. Advancement of enteral feedings was frequently interrupted by abdominal distension and emesis. Ventilation and feeding intolerance gradually improved, so that by 3 months she was at normal PEEP of +5 cm H _2_O, and tolerating 100 mL/kg/day of 0.73 kcal/mL elemental formula.

A urine bile acid profile obtained at age 2 months while on UDCA was consistent with mild-to-moderate cholestasis, but because of potential masking of a bile acid synthetic defect by UDCA, the bile acid profile was repeated at 3 months of age, off UDCA therapy for 5 days. This profile indicated a potential bile acid synthesis defect in 3β-hydroxy-Δ(5)-C(27)-steroid oxidoreductase encoded by the *HSD3B7* gene, and treatment with cholic acid (CA) was suggested at age 4 months to suppress endogenous bile acid synthesis. Pending *HSD3B7* molecular sequencing, the patient was consented under Cincinnati Children’s Hospital Study #2011-2448 to participate in an emergency compassionate use, open-label study of enteral CA (Asklepion Pharmaceuticals, Baltimore, MD, USA) under protocol #CAC-002-01. The CHOC Children’s Institutional Review Board was consulted due to her severely ill condition, and allowed emergency access for the patient to be CA contingent upon parental consent to the Cincinnati Children’s Study.

**Figure 1.**
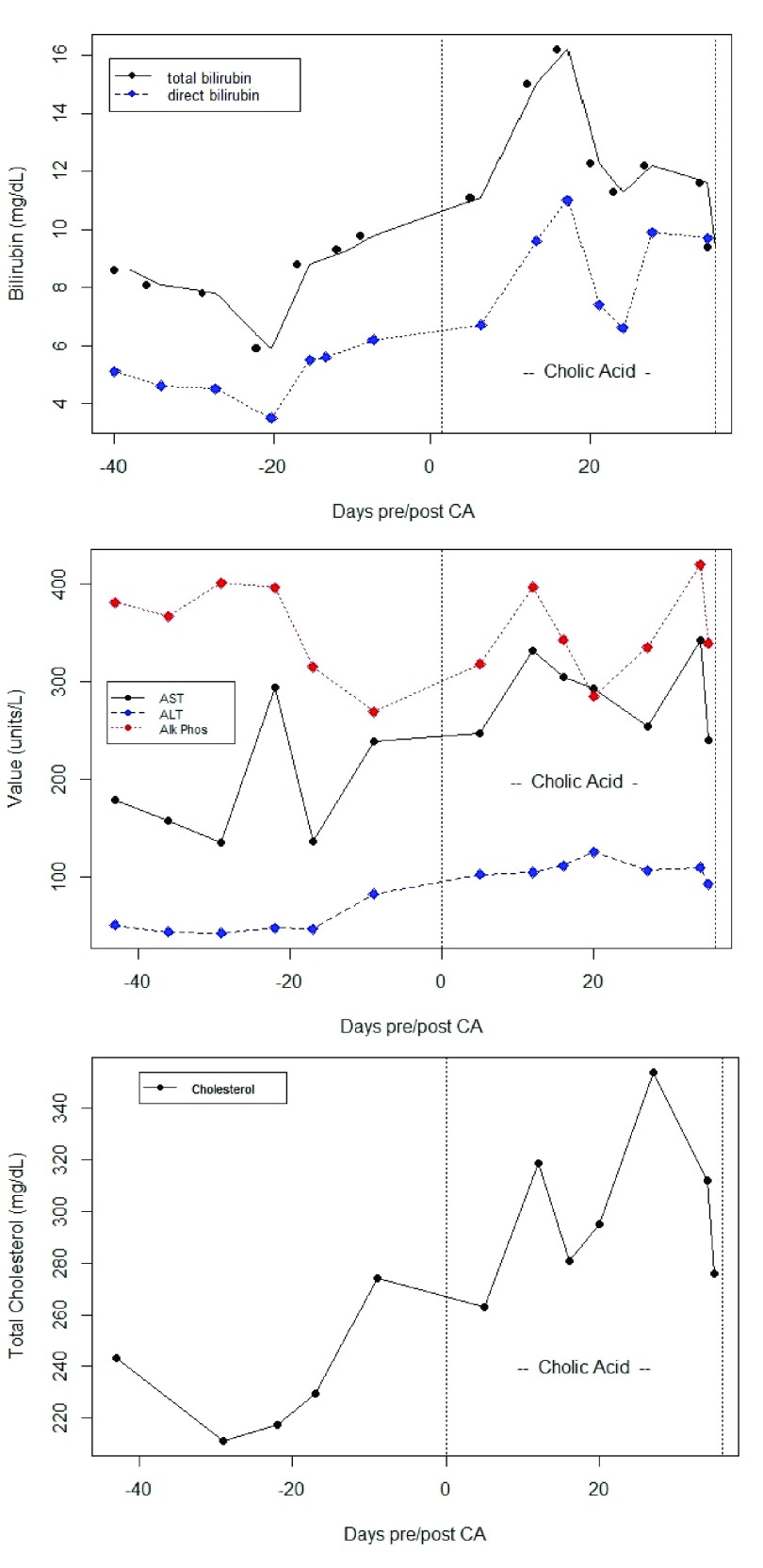
Graphs of hepatic function parameters from Case 1 as patient transitioned from ursodeoxycholic acid (UDCA) to cholic acid (CA) therapy. Note post-CA increases in bilirubin, transaminases, and total plasma cholesterol, which paralleled deterioration of the patients’ clinical status.

**Figure 2.**
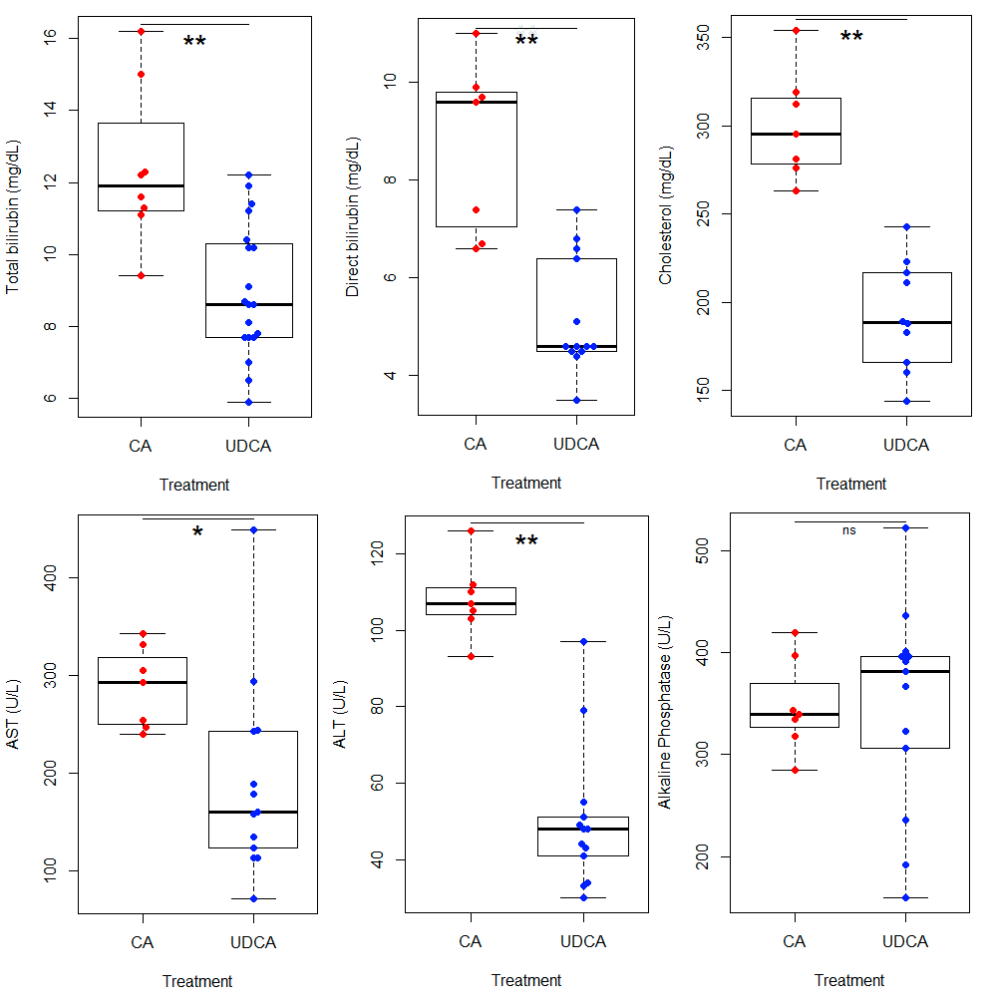
Boxplots of hepatic function parameters from Case 1. These indicate statistically significant (Student’s T-test, performed with Rv3.2.0: *, p < 0.05; **, p < 0.005) elevations in all denoted parameters except alkaline phosphatase, while on cholic acid (CA) compared to ursodeoxycholic acid (UDCA) therapy.

At 4.5 months of age, the patient began CA, 15 mg/kg/day. Her stable pre-CA elevation in transaminase and bilirubin levels began to increase 7 days after initiation of CA, and continued to increase (See Figure 1 and Figure 2). She also developed worsening hepatosplenomegaly, vomiting, and increase in abdominal girth from 39 cm pre-CA to 46 cm. Twenty-five days after CA initation, she began to have bradycardic / desaturation events without signs of infection. The levels of PEEP required to prevent bronchospasm increased from +5 to +10, and eventually +15 cm H _2_O. She remained on CA therapy for a total of 37 days; due to worsening of clinical and laboratory parameters and no mutations identified in *HSD3B7*, CA was discontinued and UDCA restarted at 30 mg/kg/day ( Figure 1 and Figure 2). She was transferred to another centre for consideration of orthotopic liver transplantation, where UDCA and high ventilator support was continued. Her PEEP was slowly titrated downwards, eventually reaching +6 cm H _2_O at age 8 months. At age 12 months, whole exome sequencing identified compound heterozygous pathogenic *NPC1* mutations (c.2213C>A / c.3234_3237dupATTT) and the decision was made not to proceed with liver transplantation due to the NPC1 diagnosis.

Subsequently, she was enrolled in an emergency compassionate use, single-patient Investigational New Drug study (CHOC Children’s study #140223) of open-label intravenous and intrathecal 2-hydroxypropyl-β-cyclodextrin (HPβCD; Janssen Research & Development). To date, she has safely received 148 intravenous treatments that started at 500 mg/kg/week and escalated to her current 2000 mg/kg/week dose by 14 weeks of treatment. Her initial intrathecal dose was 175 mg monthly, delivered via lumbar puncture, and gradually escalated to her current dose of 300 mg every other week. She has received 57 intrathecal treatments to date. Plasma cholestane-3β,5α,6β-triol dropped from 0.34 nmol/mL (pre-treatment) to 0.04 nmol/mL; RR <0.02 nmol/mL. Her latest total bilirubin was 0.6 mg/dL, AST 51 U/L, ALT 28 U/L, alkaline phosphatase 149 U/L, prothrombin time international ratio of 1.10, and fibrinogen of 251 mg/dL (all normal). Her γ-glutamyl transpeptidase is now elevated at 120 IU/L and partial thromboplastin time prolonged at 44.1 s. Her NPC Severity Score, which assesses a broad range of symptoms and level of neurologic function and increases with worsening disease ^4^, was 30 prior to initiation of HPβCD, 24 in Year 1, and 26 by Year 2. Developmentally, she is delayed, but currently sitting up without support, speaking several words, waving hello and goodbye, flipping book pages, and taking steps with assistive device support.

## Case 2

A female child of Spanish descent was born at 38 ^+6 ^weeks to a healthy non-consanguineous couple with another healthy 2-year-old daughter. Her birth weight was 2730g (9–25 ^th^ centile- UK-WHO growth charts ^22^). At 3 weeks of age she was investigated for persistent jaundice, and was found to have cholestasis with abnormal liver function tests (total bilirubin 8.5 mg/dl RR <1.2 mg/dl; direct bilirubin 5.5 mg/dl; ALT 44 U/l, RR <33 U/l; AST 201 U/l, RR 9-80 U/l; GGT 80 U/l, RR 6-42 U/l) and marked hepatosplenomegaly noted both on clinical examination and abdominal ultrasound. A bone marrow biopsy demonstrated foam cells suspicious for NPC; the diagnosis was confirmed at 4 months of age both by abnormal fillipin staining of skin fibroblasts, indicative of unesterified cholesterol storage, and identification of pathogenic compound heterozygous *NPC1* mutations (c.2978delG/c.3245+1dup).

Her liver function remained abnormal but stable (at 12 months of age: total bilirubin 0.5 mg/dl; ALT 68 U/L; AST 120 U/l; GGT 149 U/l); she gained weight following between the 2 ^nd^ and 9 ^th^ centiles. She remained free of neurological features except for general motor delay. She was commenced on miglustat at 9 months of age, at a dose of 14mg once daily titrated to 33mg three times daily over a 3-month period. She continued to take an extensively hydrolysed formula, for a co-existent cows’ milk protein allergy, vitamin D supplementation, sodium bicarbonate and the dietetic supplements dextro-maltose and medium chain triglycerides.

**Figure 3.**
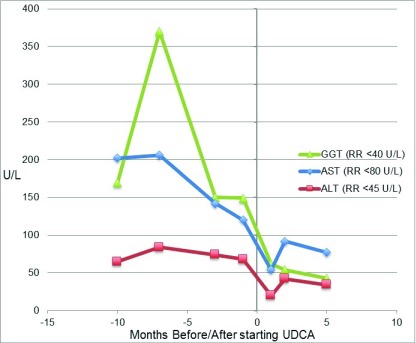
Graph of hepatic function parameters from Case 2 in the months before and after starting ursodeoxycholic acid (UDCA). This demonstrated a marked improvement in liver function parameters.

At 13 months she was commenced on UDCA at a dose of 20mg/kg/day. At review 1 month later there was a significant improvement in liver function tests (total bilirubin 0.5 mg/dl; ALT 20 U/L; AST 54 U/l; GGT 62 U/l), this improvement was sustained when further tested aged 18 months and 2 years ( Figure 3). With the commencement of UDCA, the patient’s parents also reported subjective improvements in appetite, reduced vomiting, alertness, activity levels and sleeping routine. In addition there has been an absolute and relative increase in her weight, at 15 months 8.7kg (25 ^th^ centile), maintained now at more than 2 years of age between the 25 ^th^ and 50 ^th^ centiles. She has continued on treatment with miglustat (15 mg/kg/day) and UDCA (20 mg/kg/day).

## Case 3

A male child of British descent was born at 37 ^+1^ weeks, the first child of healthy non-consanguineous parents. At birth he weighed 3374g (75 ^th^ centile UK-WHO growth charts ^22^).

At 5 weeks of age, he was noted to have cholestatic jaundice, poor weight gain and marked hepatosplenomegaly (total bilirubin 5.7 mg/dl, RR <1.2 mg/dl; direct bilirubin 4.68 mg/dl; ALT 195 U/l, RR <50 U/l; GGT 170 U/l, RR <25 U/l; Alk Phos 1257 U/l, RR 210-500 U/l). Initial investigations for neonatal cholestasis excluded infective, endocrine and surgical causes. Subsequent investigations, including white cell lysosomal enzymes, were normal, with the exception of a marginally elevated chitotriosidase level 166 (RR 4-120). A bone marrow biopsy was inconclusive, however subsequent liver biopsy demonstrated a cholestatic giant cell hepatitis with features strongly suggestive of Kupffer cell storage. He was diagnosed at 5 months of age with NPC, with abnormal filipin staining of skin fibroblasts. *NPC1* mutation analysis revealed compound heterozygosity for the pathogenic variants (c.3107C>T/c.3182 T>C).

He was supported during this neonatal phase of this disease with vitamin supplementation, high medium-chain fatty acid feeds, and UDCA 15mg/kg/daily. By the time of diagnosis at 5 months, his cholestasis had significantly improved and he was gaining weight well, UDCA was discontinued. He persisted with hepatosplenomegaly and mildly elevated liver transaminases (at 4 years of age ALT 115U/l, RR <50 U/l).

He was delayed in achieving his developmental milestones, rolling at 8 months, sitting unsupported at 9 months, pulling to stand at 13 months and walking independently at 18 months. Gross motor delay was exacerbated by relative hypotonia, pes planus and genu valgum. He was commenced on miglustat at 26 months at an initial dose of 100mg once daily, increasing to 100mg twice daily after 1 month, and then a subsequent dose increase to 200mg twice daily as per dosing guidance (British National Formulary for Children (BNFC) ^23^).

His on-going development has been notable for marked cognitive impairment, ataxia, although independently mobile, and dysarthria. Vertical supranuclear gaze palsy was first noted at 6 years. He attended a mainstream school with a 1-to-1 support and an individualised curriculum.

**Figure 4.**
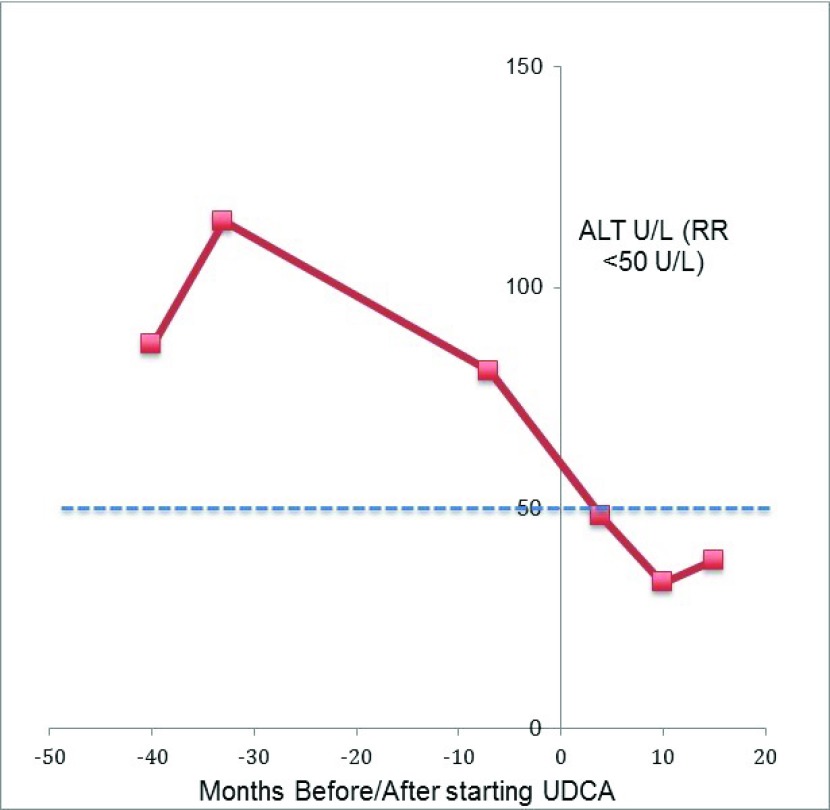
Alanine transaminase (ALT) of Case 3. This demonstrated abnormal levels before starting ursodeoxycholic acid (UDCA) that normalized following initiation of UDCA. Dotted line signifies normal value (less than 50 units/L)

**Figure 5.**
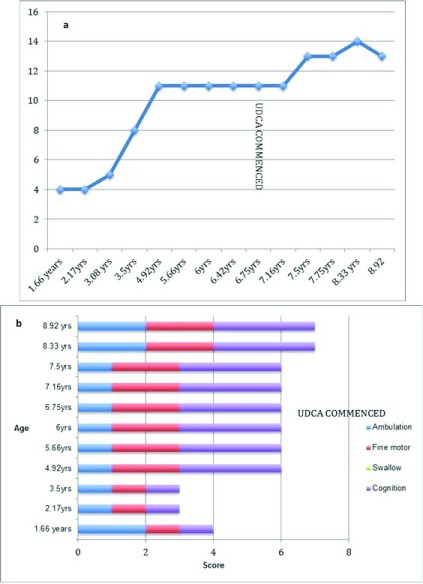
NIH scoring in Case 3. ( **a**) Total NIH score (without hearing and auditory brainstem response) for Case 3. Time of ursodeoxycholic acid (UDCA) commencement (6.67 yrs) marked on graph. ( **b**) NIH severity scale scores in the four key domains (Ambulation, Fine motor, Swallow and Cognition) for Case 3, both before and after commencement of UDCA.

At 6 years 8 months, he was recommenced on UDCA at a dose of 10 mg/kg twice daily. After commencing UDCA, the persistently elevated ALT levels returned to normal range (ALT 48 U/l, RR<50 U/l) and this has been maintained for over 2 and a half years ( Figure 4). His NIH severity score, progressed by two points over the two years since commencing UDCA, but of the four key domains, in Ambulation, by only a single point ( Figures 5a and b). At 8 years 4 months he was enrolled on the VTS-270 clinical study ^24^. His current treatments include miglustat 200mg twice daily, UDCA 10mg/kg twice daily, Glycopyrronium Bromide 2mg twice daily and *Saccharomyces cerevisae* (Brewer’s yeast) 300mg twice daily.

## Case 4

A male child of British descent was born at 40 ^+2 ^weeks, weight 3232g (25 ^th^ centile WHO-UK ^22^), the first child of healthy non-consanguineous parents. He was noted to have poor weight gain and jaundice in the early neonatal period. At 6 weeks, he was investigated for neonatal cholestasis and hepatomegaly. Initial screens, including a bone marrow aspirate and liver biopsy, were inconclusive. A further bone marrow biopsy at 7 months was suggestive of a storage disorder. Subsequent filipin staining of skin fibroblasts and genetic analysis confirmed NPC at 9 months, with compound heterozygosity of *NPC1* (c.2886_2895del/c.3182T>C). He achieved the following gross motor milestones: sitting at 6 months; crawling at 9 months; and walking at 14 months. He had general developmental delay, was noted to have a VSGP at 7 years of age and was commenced on miglustat at 8 years of age. He had steady neurological decline and started with seizures at 13 years of age, treated with Levetiracetam. At that time, he was commenced on UDCA at an initial dose of 300mg daily, and then titrated to a total daily dose of 900mg (10 mg/kg twice daily), one month after seizure onset. His routine liver function tests were normal on commencement of UDCA and have remained so since. He has now been on UDCA and Levitracetam for over 2 years. During this time there has been a decline in his mobility and slight deterioration in the frequency of his seizures. At 15 years, he was recruited onto the Arimoclomol clinical study ^25^. His current medications include daily doses: Levetiracetam 3g, UDCA 900mg (10mg/kg twice daily), miglustat 600mg, Curcumin (Super Bio- Curcumin®) 800mg, Sodium Cromoglycate 100mg.

## Discussion

The four independent cases described in this paper, different in age, disease stage, and cared for by clinicians in three different countries, collectively suggest that UDCA may have a role in the NPC therapeutic armoury. It appears to be well tolerated. In Cases 1–3 (that had elevated liver enzymes), it seemed to improve, if not normalise, abnormalities in liver transaminases, improvements that appeared to be sustained on subsequent testing. Parents report other improvements, including activity levels, alertness and appetite, suggesting that there may be further benefit for neurological aspects of the disease. Similar neurological improvements were also demonstrated in the *Npc1 ^-/-^* mouse model ^10^. This could be explained by UDCA therapy rescuing a suppressed P450 system, therby normalising both drug/xenobiotic metabolism and its role as a key detoxification system ^10^.

There are of course limitations to these findings. UDCA was given to the four patients as an uncontrolled open-label therapy. Cases 1 and 4 had additional therapeutics begun at approximately the same time, HPβCD in Case 1 and levetiracetam in Case 4. However despite this, the preclinical work and experience in these four cases demonstrates the potential utility of UDCA in NPC disease. Based on our findings, we suggest that if UDCA is commenced due to neonatal cholestasis, it should not be discontinued - as per current practice - with normalisation of the hyperbilirubinemia, but rather continued at least until liver function has normalised.

The observation that CA an alternative bile acid, was deleterious in Case 1 was in clear contrast to UDCA, that was well tolerated in all four cases. This was further explored in a *Npc1 ^-/-^* mouse where it was found to be similarly poorly tolerated, leading to a significant increase in the liver wet weight ^26^. Interestingly in bacteria, bile acids have been suggested to be substrates for transport by orthologues of the NPC1 protein ^13^. This gives a possible explanation for CA’s deleterious effect in NPC, as CA (more hydrophobic than UDCA) would accumulate in the mammalian lysosome due to loss of NPC1 function, and thereby contribute to the liver hypertrophy seen in the mouse model.

Taken together, our findings of the benefits of UDCA in NPC patients needs to be verified in a controlled clinical trial involving a larger number of NPC patients, to ascertain the likely magnitude and breadth of the effects, the benefit/risk profile and the appropriate timing of its use. CA should be considered contraindicated in NPC disease based on Case 1 and the observation of liver hypertrophy in the *Npc1 ^-/-^* mouse.

UDCA certainly has the potential to be an attractive therapy. It was well tolerated in all four cases and is the first treatment evaluated in NPC patients that address NPC-associated liver disease, which has no current therapy. Furthermore, UDCA is a licensed, readily available, safe and affordable drug making this a promising candidate to form part of a combination therapy approach for NPC disease management in the future.

## Consent

Written informed consent for publication of the patients’ details was obtained from the parents of the patients.

## Data availablity

Raw data ‘UDCA in NPC’ is available on OSF ( http://doi.org/10.17605/OSF.IO/4CGDT ^27^)
