# Supplementary material for: Case Report: Ursodeoxycholic acid treatment in Niemann-Pick disease type C; clinical experience in four cases
Source: Wellcome Open Res. 2017 Aug 31;2:75. [Version 1] doi: 10.12688/wellcomeopenres.11854.1 (PMC5649119; doi:10.12688/wellcomeopenres.11854.1)
Supplement: Peer review history

# Peer review: Referee response for version 1

**DOI:** 10.21956/wellcomeopenres.12809.r26787

**Published:** 2017-10-19

**Authors:** Jayesh Sheth

**Affiliations:** 1 FRIGE’s Institute of Human Genetics, FRIGE House, Gujarat, India

Niemann-Pick disease type C (NPC) is a fatal, neurodegenerative, intracellular cholesterol storage disorder. Recent studies have shown that three unknown bile acids that are increased in plasma from NPC but not control subjects form the base of this study. In *Npc1 ^-/-^* mice model, reduced activity of P450 enzymes was observed possibly as a result of bile acid deficiency/imbalance, as bile acid treatment significantly rescued P450 enzyme activity in *Npc1 ^-/-^* mice. Based on this present study by William and his group have shown beneficial effect of UDCA by improving liver function in three patients and improvement of activity levels. Alertness in parents. This can be further elaborated The UDCA may be of use in the improvement of neurological dysfunction in other storage disorders and need careful study.

 Authors have demonstrated the new concept of improving suppressed hepatic cytochrome 450 utilizing UDCA in patients with NPC1. The said compound is used in patients with cholestatic disorders like PBC with a rational that long term use of UDCA changes the composition of endogenous bile acid pool and further showing the beneficial effect in mouse model with NPC1. The study is based on the effect of UDCA through different mechanisms like protection of cholangiocytes against cytotoxicity of hydrophobic bile acids, reduction of bile acid cytotoxicity of bile and, possibly, decrease of the concentration of hydrophobic bile acids in the cholangiocytes, stimulation of hepatobiliary secretion, putatively via Ca(2+)- and protein kinase C-alpha-dependent mechanisms and/or activation of p38(MAPK) and extracellular signal-regulated kinases (Erk) resulting in insertion of transporter molecules (e.g., bile salt export pump, BSEP, and conjugate export pump, MRP2) into the canalicular membrane of the hepatocyte, protection of hepatocytes against bile acid-induced apoptosis, involving inhibition of mitochondrial membrane permeability transition (MMPT), and possibly, stimulation of a survival pathway.

 The only limitation of the study is authors have not shown bile acid concentration that could have completely explained the beneficial effect of UDCA rescusing suppressed P450 system normalising drug metabolism and its role as a key detoxification system in many other liver disorders besides - primary biliary cirrhosis (PBC) and primary sclerosing cholangitis (PSC).

 It is also important to study whether same mechanism can be extended for NPC2 patients where pulmonary involvement is more commonly seen together with liver dysfunction.

 More clinical trials are needed globally as a multicentric clinical trial before UDCA is approved as a potential therapeutic agent in NPC1 patients.

 All authors deserve compliments for this important research work to the rescue of rare disease patients.

I have read this submission. I believe that I have an appropriate level of expertise to confirm that it is of an acceptable scientific standard.

# Peer review: Referee response for version 1

**DOI:** 10.21956/wellcomeopenres.12809.r26287

**Published:** 2017-09-26

**Authors:** Maria Dolores Ledesma

**Affiliations:** 1 Center for Molecular Biology 'Severo Ochoa', Autonomous University of Madrid-Spanish National Research Council, Madrid, Spain

This clear and well-organized study reports the effect of bile acid therapy using the Ursodeoxycholic acid (UDCA) in four independent patients of Niemann Pick disease type C (NPC). This therapeutic strategy finds support on recently published data showing the link between an imbalance in bile acid synthesis and the impairment of the P450 detoxification system in the liver of NPC animal models. The study supports several valuable conclusions:

- UDCA treatment improves liver function
- There may also be benefit for neurological aspects of the disease
- UDCA treatment was well tolerated by all patients
- In contrast, the use of cholic acid showed deleterious effects

 

 As the authors state, the reduced number of patients analyzed and the uncontrolled open-label therapy that included different additional therapeutics, limits the significance of the study. Still the benefits found, common to all four cases with different age, disease stage and care given in different countries, strongly support the utility of UDCA in NPC-associated liver disease. This report encourages a controlled clinical trial for UDCA involving a larger number of NPC patients that would detail UDCA treatment conditions for liver disease and confirm the potential neurological effects. Given that NPC-associated liver disease has no current therapy and that UDCA is a licensed, safe and affordable drug the results here shown are useful for the NPC community and worth sharing.

I have read this submission. I believe that I have an appropriate level of expertise to confirm that it is of an acceptable scientific standard.
